# Rare Case of Non-Keratinizing Squamous Cell Carcinoma in the Lacrimal Sac Treated with Surgery

**DOI:** 10.3390/jcm13154395

**Published:** 2024-07-27

**Authors:** Jakub Pośpiech, Julia Hypnar, Grzegorz Horosin, Kamil Możdżeń, Agnieszka Murawska, Maria Przeklasa, Marcin Konior, Jerzy Tomik

**Affiliations:** 1 Faculty of Medicine, Jagiellonian University Medical College, 31-008 Cracow, Poland; 2 Faculty of Dental Medicine, Jagiellonian University Medical College, 31-008 Cracow, Poland; 3 Department of Otolaryngology, Jagiellonian University Medical College, 30-688 Cracow, Poland

**Keywords:** lacrimal sac tumor, lacrimal sac malignancy, squamous cell carcinoma, non-keratinizing SCC, orbital tumor, dacryocystectomy, reoperation, recurrence

## Abstract

**Background and Objectives**: The objective of this study was to present a rare case of non-keratinizing squamous cell carcinoma (SCC) of the lacrimal sac (LS). Neoplasms of the lacrimal drainage system are extremely rare. These lesions are predominantly malignant and are associated with a high mortality rate. **Case Presentation:** A 51-year-old woman was referred to an ophthalmologist with a history of unilateral epiphora, presbyopia, and abnormal eye discharge. Antibiotic therapy was applied and modified later due to persisting symptoms. After five months, edema of the medial left eye angle occurred. A lacrimal sac incision was performed, and a subsequent magnetic resonance imaging (MRI) scan revealed a 2 cm, contrast-enhanced solid tumor. The patient was qualified for dacryocystectomy, which was conducted by the otolaryngology department. Postoperative histopathologic findings indicated the presence of non-keratinizing SCC. During a reoperation, margins were extended, and the surgery was found to be radical. Following the reoperation, no alarming symptoms were observed. However, a follow-up MRI and positron emission tomography (PET) scan six weeks later revealed metastases. Further treatment is being planned. **Conclusions:** LS tumors are life-threatening conditions that are challenging to diagnose at an early stage. Surgical excision is the preferred treatment option. Imaging studies play an important role in post-operative follow-up because of the possibility of recurrence and metastasis, even after radical surgery.

## 1. Introduction

Tumors of the lacrimal sac (LS) are rare occurrences [1]. They are classified as tumors of the orbit and its adnexa [2], or as secondary orbital tumors [3]. Tumors of the orbital region have an incidence of less than one case per million people per year [4]. Studies involving thousands of orbital tumors have reported single cases of LS tumors [2,3,5]. Since the late 18th century, it is estimated that there have been approximately 800 LS tumors [6].

In the entire lacrimal drainage system, LS is the most common site of neoplasms. The majority of LS tumors are malignant and originate in the epithelium [7,8]. The most prevalent malignant neoplasm is squamous cell carcinoma (SCC), while non-keratinizing SCC is a considerably less frequent occurrence [6,9].

The cause of LS tumors is uncertain. Risk factors include human papillomavirus and Epstein–Barr virus infections, as well as chronic inflammation [6,9]. Malignant neoplasms typically develop in the fifth decade of life, occurring later than benign lesions [6,7,8,9,10]. While non-keratinizing SCCs affect both sexes equally, SCCs of the LS are slightly more common in men [9].

Malignant epithelial tumors, in approximately 70% of cases, present with unilateral epiphora lasting for several months and/or with a mass in the LS region [6,9]. Symptoms, such as pain or blood-stained epiphora, are less common. They occur in 4–10% of cases but indicate an advanced stage of the neoplasm [6,9]. A considerable number of tumors advance without presenting characteristic symptoms and are incidentally detected during other surgical procedures [6,8].

Neoplasms of the LS can be life-threatening, with mortality rates ranging from 10% to 40% [6,7,8]. Early diagnosis and prompt initiation of therapy are crucial for improving the chances of a cure [7,8]. Surgical resection is the primary treatment, while the inclusion of radiotherapy and/or chemotherapy is determined based on the histologic subtype and the radicality of the tumor excision [6,7,9].

The aim of this study is to provide clinically valuable information for the diagnosis and detailed surgical and pharmacological management of a rare non-keratinizing SCC resected from the LS.

## 2. Case Presentation

**Figure 1:**
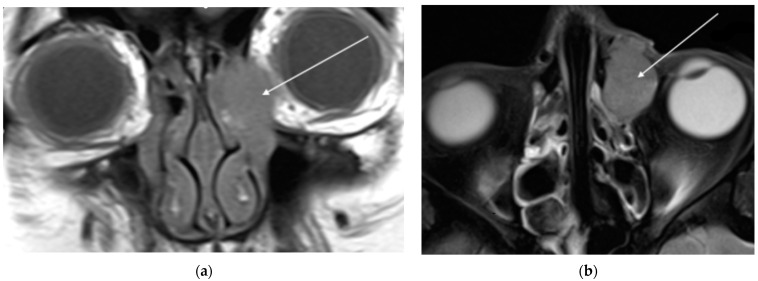
The MRI T1 (**a**); and T2 (**b**) projections of a 51-year-old woman demonstrate a solid, lesion in her left lacrimal sac fossa, indicated by the white arrow.

A 51-year-old woman with no comorbidities presented to an ophthalmologist with presbyopia and excessive lacrimation of the left eye for approximately one month. The ophthalmic examination revealed foamy mucus and a papillary reaction of the conjunctiva, which was treated with azithromycin eye drops. At the 4-month follow-up visit, the patient’s symptoms persisted, leading to further antibiotic therapy with erythromycin and ofloxacin drops. Five months after the primary consultation, the patient was admitted to the ophthalmology department with edema of the medial angle of the left eye and mild (3/10) left ocular pain radiating to the forehead and maxilla. During hospitalization, the lesion was incised, but no tissue sample was taken for histopathology. Head magnetic resonance imaging (MRI) was performed. The MRI revealed a solid, well-demarcated lesion, measuring 24 × 14 × 25 mm, located in the left LS fossa (Figure 1). The finding showed moderate enhancement after contrast and moderately restricted diffusion. The tumor mass extended into the dilated left lacrimal canal and ethmoidal bulla. The left middle nasal concha and left orbit were, consequently, modulated. No abnormal lymph nodes were observed.

The following month, the patient was again hospitalized in the specialized otolaryngology department and qualified for the surgical treatment. Dacryocystectomy was performed using both endoscopic and external approaches. The procedure included medialization and partial resection of the left middle nasal concha, as well as removal of the anterior and posterior ethmoid mucosa. Additionally, the opening of the left maxillary sinus was widened. Furthermore, part of the lacrimal bone and the frontal process of the maxilla were resected, exposing the LS. A solid tumor in the projection of the lacrimal ducts was then exposed and fully dissected, reaching the endoscopically made wide drainage.

Post-operative histopathologic tests revealed carcinoma planoepitheliale akeratodes male differentiatum G3—infiltration carcinomatosa, microscopically present within the surgical margins (R1). The immunohistochemical profile demonstrated positivity for cytokeratines (CK), CK5/6, p53 and p40, but was negative for human melanoma black-45 (HMB-45) and leukocyte common antigen (LCA). There was no sign of mucus secretion in any of the examined samples.

Regarding the positive resection margins (R1), the patient was discussed again and qualified for reoperation, which was performed 3 weeks later. The resection margins along the lacrimal ducts were extended peripherally, using the external approach. Postoperative endoscopic observation revealed no residual lesions. Histological analysis of the specimens taken during the procedure, including the scar of the left suborbital skin region, the lacrimal fossa, and the uncinate process of the ethmoid bone regions, showed the absence of cancerous tissue.

At the follow-up visit two weeks after the reoperation, the patient’s results were found to be within the normal range with no alarming symptoms. Six weeks later, a control MRI examination was conducted, which revealed the presence of multiple metastatic lymph nodes. A positron emission tomography (PET) scan confirmed the presence of metastases in the cervical lymph nodes group IIA and IB on the left side. Additionally, the scan indicated the presence of a neoplasm with metabolic activity in the left lacrimal sac that adhered to the left eye laterally and the nasal septum medially; the inferior border of the neoplasm was in the maxillary sinus.

The patient was consulted with by both the oncology and otolaryngology departments. Consequently, the patient decided to undergo chemotherapy with four courses of 175 mg cisplatin infusions with adjuvant proton beam therapy.

## 3. Discussion

LS tumors are extremely rare. Shields et al. [3] conducted a study on patients diagnosed with orbital tumors in which, out of 1264 patients enrolled in the study, only 2 had tumors that originated in the LS. Scat et al. [2] provided a significant epidemiological study of 1705 malignant tumors of the eye and adnexa. In this study, only two of the tumors were located in the LS. A recent major review on LS tumors conducted by Ramberg et al. [9], performed through database searches in the literature from 1960–2019, consisted of only 539 cases. A few case reports on patients with SCC have been published to date [1,10]. A comparison of cases found in the literature is provided in Table 1.

LS tumors can be classified as primary, secondary, or metastatic. A primary tumor is defined as a neoplastic growth that originates at the site of the initial tumor development and subsequently forms a malignant mass. While the majority of solid cancers originate at their primary site, they may subsequently metastasize or spread to other parts of the body. This process results in the formation of secondary tumors. The majority of cases of LS tumors are primary epithelial tumors. Malignant tumors are more common than benign ones [6,7,8,9,10]. SCC is the most prevalent type (55%), followed by non-keratinizing SCC (20%), mucoepidermoid carcinoma (MEC) (8%), adenocarcinoma (6%) and adenoid cystic carcinoma (4%). Secondary tumors can spread to the LDS from distant locations, or, more commonly, grow into the LDS from neighboring locations such as the skin, eyelids, conjunctiva, orbits, or the sinonasal cavity [9].

The etiopathology of LS tumors is yet to be discovered. SCC may develop de novo or in a pre-existing papilloma. The risk factors include chronic inflammation, and HPV and EBV infections. Histological risk factors for malignant transformation include increased nuclear atypia and mitotic activity, HPV 6/11 or HPV 16/18 positivity, overexpression of p53, epidermal growth factor receptor (EGFR), and transforming growth factor-alpha (TGFα) [6]. LS malignant tumors usually occur in older patients, with the highest prevalence in the 50- to 60-year age group; benign tumors are often diagnosed a decade earlier [8]. Some studies show a slight male predominance [9]. The others indicate equal frequency in both sexes [6].

The initial symptoms of an LDS carcinoma include epiphora and dacryocystitis; therefore, the clinical presentation at early stages might resemble those of chronic dacryocystitis [6,7,8,9,10,11]. This might lead to a delay in the proper diagnosis and implementation of the therapeutic approach at early stages. To avoid this mistake, which may lead to progression of the tumor, it is necessary to perform a biopsy [6,9].

At more advanced stages, clinical presentation tends to be less misleading, with a palpable mass in the LS region as a predominant symptom. Kuo et al. [11] provided a significant amount of information on the topic, conducting a study on 65 patients diagnosed with LS tumors, which is a remarkable number of cases considering the rarity of the syndrome. This study provides comparisons of various symptoms experienced by patients diagnosed with benign tumors, primary malignant tumors, and secondary malignant tumors. The most common presenting symptoms in all the groups were a palpable lump/mass and epiphora. Bloody tears were observed in 5% of the benign group and in 20% of the malignant group. A palpable mass extending above the medial canthal tendon was noted in 9% of the benign group and in 74% of the malignant group, respectively, and could be useful in differentiating between the groups. Some patients with secondary malignancy also presented with nasal obstruction, epistaxis, tinnitus, or otalgia [11].

Our patient was a 51-year-old woman with no risk factors indicating an LDS carcinoma. Initially, the only symptom she presented was excessive lacrimation of the left eye; therefore, antibiotic treatment was initiated on suspicion of dacryocystitis. Five months later, the manifestation of further symptoms, including edema of the medial angle of the left eye and mild left ocular pain, prompted investigation of a potential LDS malignancy. The head MRI was performed, which revealed a solid, well-demarcated lesion located in the left LS fossa and extending into the dilated left lacrimal canal and ethmoidal bulla. The left middle nasal concha and left orbit were likewise occupied.

Diagnostic imaging procedures are essential for identifying malignant lesions in LDS. Dacryocystography (DCT), computed tomography (CT) and MRI are most frequently used in identifying LS tumors [1,6,9,10]. DCT can only be used to identify a space-occupying mass within the LDS. CT and MRI provide information about the characteristics of the neoplasm [9]. CT scans are more sensitive than MRI for the evaluation of bone erosion, which is highly suggestive of malignancy. Nonetheless, MRI is superior to CT in detecting early invasion of the surrounding tissue, as it enables the differentiation of inflammatory lesions from malignant ones. Furthermore, MRI is superior to CT in assessing local extension into the soft tissues and orbit [6]. In our case, only the MRI was performed, which revealed a lesion in the left LS fossa extending into the dilated left lacrimal canal and ethmoidal bulla. The left middle nasal concha and left orbit were also occupied.

The management of LS tumors is based on the histopathological diagnosis, the size and extension of the lesions, occupation of the lymph nodes, and distant metastasis. In our case, the initial lesion was incised without the acquisition of a histopathological sample. Instead, an MRI scan was conducted, providing valuable information about the location and extent of the lesion. The image obtained was characteristic of malignant lesions; therefore, the absence of a histopathological confirmation of the carcinoma did not significantly alter the planned extent of the operation.

The available treatment methods for LS tumors include surgery, radiotherapy, and chemotherapy [6,9]. Benign tumors mostly require only excision of the lesion with DCR or DCT. Extensive malignant lesions might require a more invasive operation, including removal of the canaliculi, ethmoidectomy, maxillectomy, or orbital exenteration. Following the procedure, a histological evaluation of surgical margins should be conducted to ensure complete removal of the tumor [6,9,10]. Additionally, adjuvant radiotherapy is recommended for histologically aggressive subtypes and for T3–T4 staged tumors according to the AJCC classification. It should be also considered in patients with stage T2 tumors. Furthermore, it should be recommended in cases of narrow or positive margins. Chemotherapy is mostly used as a palliative treatment for patients with unresectable tumors or patients with distant metastasis [9].

Definitive radiotherapy is indicated for patients who refuse surgical treatment or who have unresectable tumors. Song et al. [1] described a significant cohort of 17 patients with LSSCC. In this study, all the patients were treated with definitive radiotherapy, and 11 patients received cisplatin-based chemotherapy. None of the patients received surgical treatment because of unresectable lesions or refusal to have surgery.

In our case, based on the extent of pathologic lesions, the decision to perform dacryocystectomy (DCR) using both an endoscopic and external approach was made. The procedure included medialization and partial resection of the left middle nasal concha, removal of the anterior and posterior ethmoid mucosa, and widening of the left maxillary sinus opening. Furthermore, the lacrimal bone and frontal process of the maxilla were partially resected. A solid tumor in the projection of the lacrimal ducts was then exposed and fully dissected, reaching the endoscopically made wide drainage. Post-operative histopathological tests revealed low-grade (GIII) SCC with immunohistochemical positivity for CK, CK5/6, p53 and p40. When recording the histopathological findings, the patient was discussed again and qualified for reoperation, in which resection margins along the lacrimal ducts were extended peripherally using the external approach. Postoperative endoscopic observation revealed no residual lesions.

De Stefani et al. [12] described a similar case of a patient with an SCC LS tumor adjacent to the distal insertion of the medial rectus muscle but without invasion of the ethmoidal cells. The patient underwent a wide local incision, including en bloc removal of the lacrimal bone and the anterior portion of the ethmoid bone. Furthermore, a limited maxillectomy was performed. The margins of the endo-orbital extensions appeared negative for neoplastic cells, and postoperative radiotherapy was indicated.

Based on the literature, the prognosis for LS tumors is poor, as recurrence rates range from 20% to 50% and mortality rates range from 10% to 40%. Moreover, both distant and local metastases are frequently observed. Ni et al. [13] reported regional lymph node metastases in 27% of 74 malignant epithelial lacrimal sac tumors. In our case, despite the total tumor excision, metastases in the lymph nodes were found six weeks after the procedure. Early diagnosis, followed by the initiation of therapy and long-term follow-up, are crucial for improving the prognosis. Furthermore, full resection of the tumor with clean margins is significant [6,7,8,9].

**Table 1:** A comparison of the age of diagnosis, treatment, and prognosis of lacrimal sac carcinomas in the literature.

Author	SCC (n ^1^)	Mean Age of Diagnosis	Symptoms (n)	Surgical Strategy (n)	Definitive Rx (n)	Prognosis (n)
Syeed et al., 2022 [10]	2	63	watering (2), swelling (2), painless lesion in the right lacrimal sac area (1)	FNAB ^2^ followed by DCT ^3^ (1), incision biopsy (1)	EBRT ^5^ (2)	lymph node metastasis at diagnosis (1)
A De Stefani, 1998 [12]	1	60	epiphora, pain at the medial side of the right orbit	DCT followed by en bloc removal of the lacrimal bone, the anterior portion of the ethmoid bone and limited maxillectomy	EBRT	N.M.
Katircioglu et al., 2015 [14]	1	40	recurrent dacryocystitis, epiphora, bloody discharge, palpable mass within the medial canthal area	FNAB followed by medial obitotomy with en bloc excision of the tumor	EBRT	N.M.
Rahangdale et al., 1995 [15]	1	48	painless mass in the right medial orbital canthus, dacryocystitis	open biopsy followed by orbital exenteration, lateral rhinostomy, and a radical right maxillectomy	EBRT	no recurrence in 2-year follow-up
Bonder et al., 1983 [16]	1	68	tearing and mass in the right lacrimal sac region	DCT with a diagnostic biopsy	N.M.	N.M.
Song et al., 2020 [1]	69	52	epiphora (7), palpable mass (7), nosebleed/nasal obstruction (4)	N.M. ^4^	Chemotherapy (11), IMRT ^6^ (11), CRT ^7^ (6)	2-year OS ^8^ 94.1% 5-year OS 84.7%,

^1^ number of patients; ^2^ fine-needle aspiration biopsy; ^3^ dacryocystectomy; ^4^ not mentioned; ^5^ external beam radiation therapy; ^6^ intensity modulated radiation therapy; ^7^ conventional radiation therapy; ^8^ overall survival.

## 4. Conclusions

LS tumors are rare occurrences associated with a poor prognosis. At the initial stage, they may resemble recurrent dacryocystitis; therefore, it is important for clinicians to approach such cases with a high degree of suspicion. CT, MRI and DCT are the imaging modalities used to identify LS tumors. DCT with tumor excision remains the surgical strategy of choice. It is crucial to assess the histological evaluation of surgical margins after the procedure to ensure complete removal of the tumor. Other treatment options include adjuvant radiotherapy, chemotherapy, or definitive radiotherapy. Our case report indicates that even when an LS tumor is diagnosed relatively early, its course may be aggressive. The importance of imaging studies in postoperative follow-up should also be emphasized. In this case, despite the absence of lymph node involvement on the preoperative MRI, the histopathologic radicality of the second surgery, and no alarming symptoms postoperatively, lymph node metastasis was revealed. Only with the use of imaging studies was it possible to quickly introduce further therapy.

## Data Availability

The data presented in this study are available on request from the corresponding author due to privacy and legal restriction.
